# Supplementary material for: Size-dependent vitrification in metallic glasses
Source: Nat Commun. 2023 Aug 4;14:4698. doi: 10.1038/s41467-023-40417-4 (PMC10403508; doi:10.1038/s41467-023-40417-4)
Supplement: Supplementary file 1 — Supplementary Information [file 41467_2023_40417_MOESM1_ESM.pdf]

Supplementary Information for ”Size dependent vitrification in metallic glasses”

Valerio Di Lisio[1], Isabella Gallino[2,*], Sascha S. Riegler[2], Maximilian Frey[2], Nico Neuber[2], Golden Kumar[3], Jan Schroers[4], Ralf Busch[2] & Daniele Cangialosi[1,3,*]

[1] *Donostia International Physics Center, Paseo Manuel de Lardizabal 4, 20018 San Sebastián, Spain*

[2] *Saarland University, Chair of Metallic Materials, 66123 Saarbrücken, Germany*

[3] *University of Texas at Dallas, Department of Mechanical Engineering, Richardson, USA*

[4] *Yale University, Mechanical Engineering and Materials Science, New Haven, USA*

[5] *Centro de Física de Materiales (CSIC–UPV/EHU) Paseo Manuel de Lardizabal 5, 20018 San Sebastián, Spain*

email: daniele.cangialosi@ehu.eus, i.gallino@mx.uni-saarland.de

Supplementary Table 1. Sample mass and geometric parameters.

Sample composition in at.% (Flash DSC sensor/model)	Mass, m (ng)	Volume, V (μm^3)	Surface area, A (μm^2)	$l_{eq} = V/A$ (μm$\pm$0.1)	q_h (Ks^{-1})
$Au_{49}Cu_{26.9}Si_{16.3}Ag_{5.5}Pd_{2.3}$ (UFS/Flash DSC 1)	1450	110500	19200	**5.7**	1000
	71	5400	1490	**3.6**	2000
	19	1450	540	**2.7**	2000
	10	760	505	**1.5**	5000
	3	215	240	**0.9**	5000
$Au_{49}Cu_{26.9}Si_{16.3}Ag_{5.5}Pd_{2.3}$ (HTS/Flash DSC 2+)	75	5700	1570	**3.6**	300
$Pt_{57.5}Cu_{14.7}Ni_{5.3}P_{22.5}$ (HTS/Flash DSC 2+)	2100	139200	13000	**10.8**	100
	100	6535	1691	**3.9**	1000
	33	2144	804	**2.7**	1000
	9	523	314	**2.0**	2000
	4	310	222	**1.3**	2000

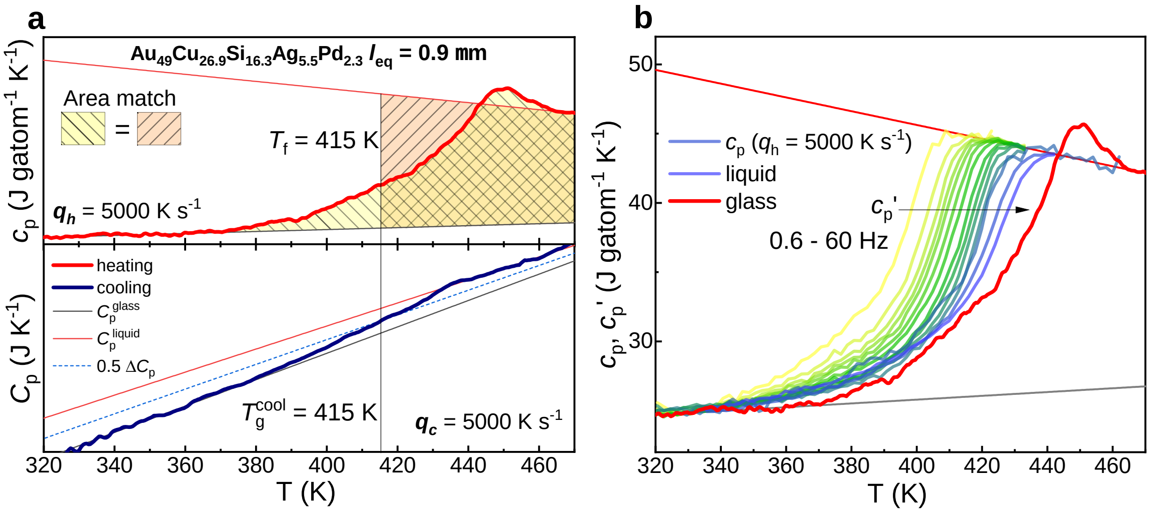

Supplementary Figure 1. Comparison between fictive temperature and glass transition on cooling; determination of glass and liquid c_p. a) Heating scan (upper panel) and previous cooling scan (lower panel), both recorded at a rate of 5000 K s^{-1} using the $Au_{49}Cu_{26.9}Si_{16.3}Ag_{5.5}Pd_{2.3}$ sample with $l_{eq} = 0.9$ μm. The fictive temperature T_f was evaluated by the Moynihan area matching method (see Supplementary Note 1) and the glass transition temperature on cooling was taken at the half step of specific heat. b) Determination of the glass and liquid c_p by combining the frequency dependent c_p' taken from step-response experiments (see inset of Fig. 1a of the main manuscript) with the c_p heating curve of panel (a). In this way, liquid c_p data were retrieved in a wide interval, ranging from 410 K up to 470 K. These liquid and glass c_p are also reported in upper panel (a) of the figure.

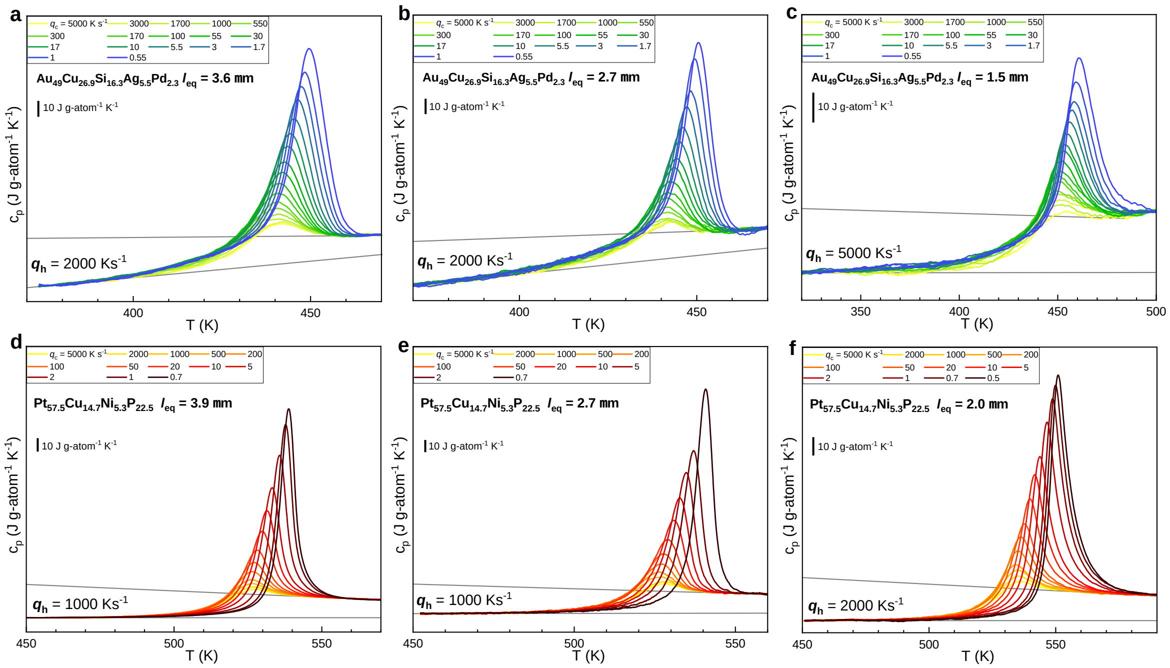

Supplementary Figure 2. Vitrification kinetics of intermediate size specimens. Specific heat scans at a heating rate q_h after cooling at various rates q_c for $Au_{49}Cu_{26.9}Si_{16.3}Ag_{5.5}Pd_{2.3}$ at. % with characteristic length l_{eq} of 3.6 μm (a), 2.7 μm (b) and 1.5 μm (c), as well as for $Pt_{57.5}Cu_{14.7}Ni_{5.3}P_{22.5}$ at.% with l_{eq} of 3.9 μm (d), 2.7 μm (e), 2.0 μm (f). The grey lines are linear fits of the specific heat of the glass and the supercooled liquid, respectively. All scans exhibit an excess endotherm in proximity of the glass transition growing in intensity as the cooling rate is decreased. This indicates the achievement of lower enthalpic states as the sample experiences slower cooling.

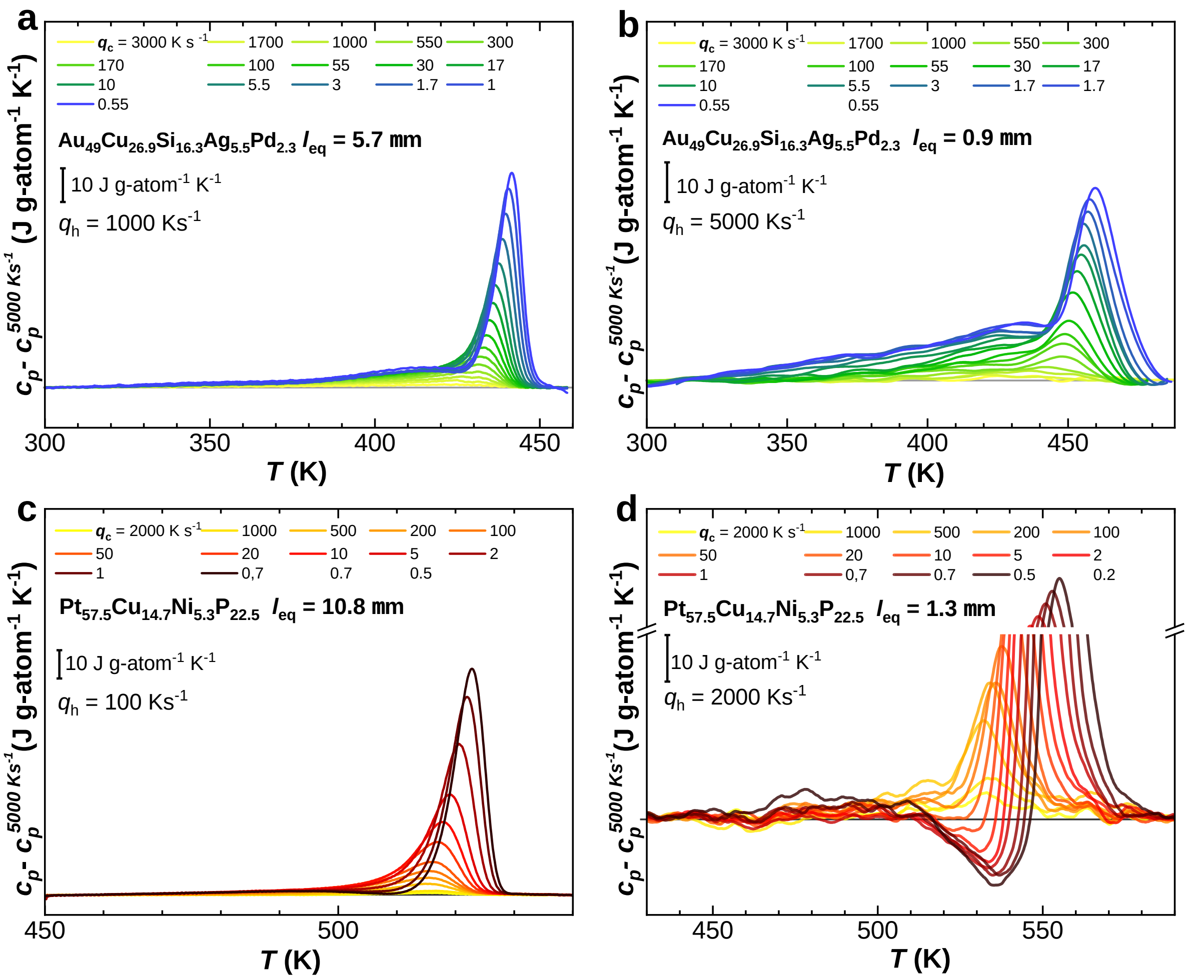

Supplementary Figure 3. Excess specific heat curves as a result of cooling at various rates. Excess specific heat between scans after cooling at the indicated q_c rates and the fastest cooling at 5000 K s^{-1}. The grey lines are guides for the eye to indicate the zero excess. $Au_{49}Cu_{26.9}Si_{16.3}Ag_{5.5}Pd_{2.3}$ at. % glass exhibits a clear broad endotherm between 320 and 430 K, growing in intensity with decreasing sample size from l_{eq} = 5.7 μm (a) to 0.9 μm (b). This feature, though present, is less pronounced for both $Pt_{57.5}Cu_{14.7}Ni_{5.3}P_{22.5}$ at.% specimens with l_{eq} = 10.8 μm (c) and 1.3 μm (d).

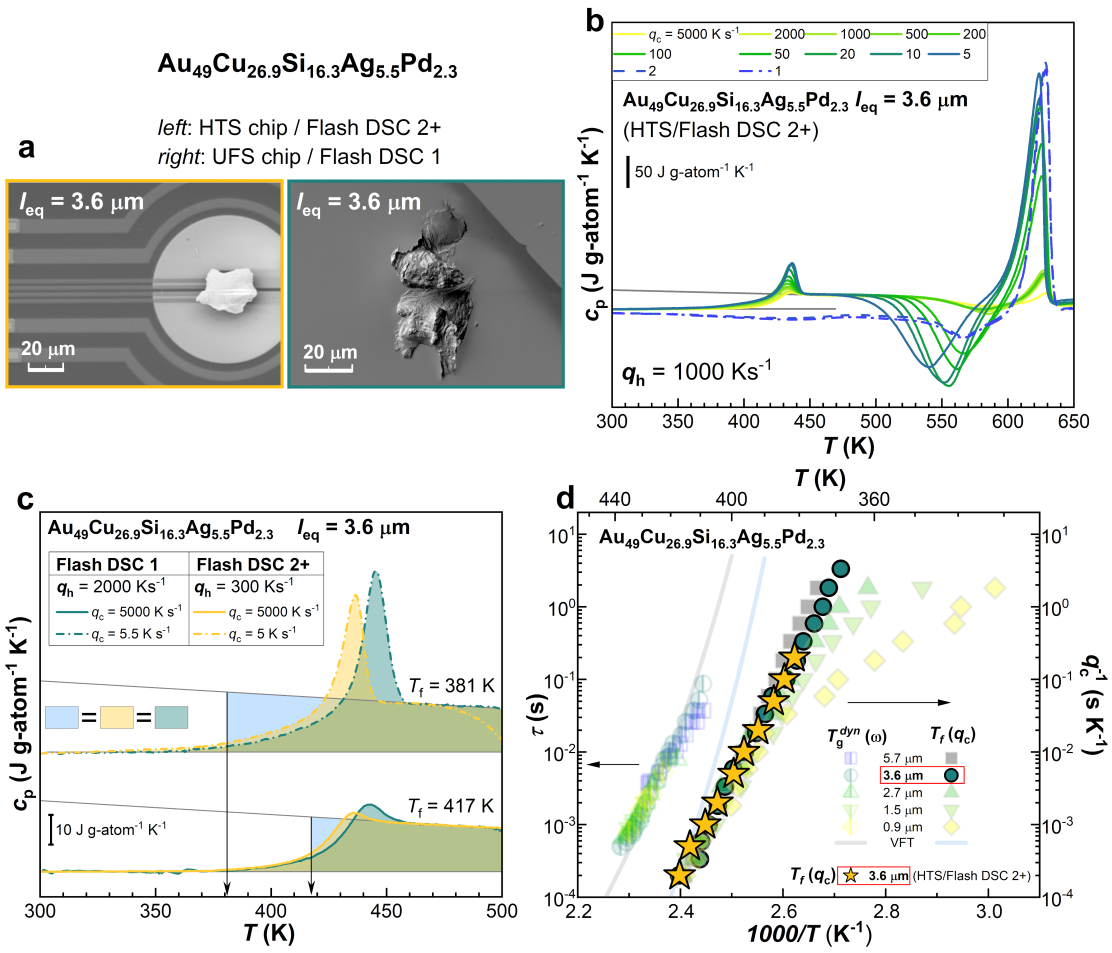

Supplementary Figure 4. Comparison between $Au_{49}Cu_{26.9}Si_{16.3}Ag_{5.5}Pd_{2.3}$ at. % glasses with different geometries. Study of vitrification kinetics in samples with identical equivalent size. (a) SEM images of samples with identical l_{eq} deposited on the chip for Flash DSC 2+ (left panel) and on that for Flash DSC 1 (right panel). (b) Specific heat scans acquired with the Flash DSC 2+ with the $Au_{49}Cu_{26.9}Si_{16.3}Ag_{5.5}Pd_{2.3}$ at. % bearing l_{eq} = 3.6 μm and a sphere-like geometry. The scans are obtained at the given q_H after cooling at various q_c. (c) Comparison of heat flow rate scans after cooling at two rates of samples with identical size and film-like sample (measured by Flash DSC 1) and the irregularly shaped sphere sample (measured by Flash DSC 2+) (d) Comparison

between vitrification kinetics of equal equivalent size samples, namely the film-like sample (green circles corresponding to Flash DSC 1 data) and the irregularly shaped sphere sample (yellow stars corresponding to Flash DSC 2+ data). The additional shadowed data are the same as in Fig. 3a of the main manuscript.

Supplementary Notes

Supplementary Note 1. Evaluation of fictive temperature and comparison with the glass transition temperature on cooling. The cooling rate dependency of the fictive temperature, T_f, was evaluated by means of the Moynihan method (Ref. (86) of the main manuscript). The temperature protocol started with the melting of the specimen well above its liquidus temperature, followed by an isotherm of 0.1 s and cooling at q_c = 5000 K s^{-1} down to 443 K for the Au-based glass-former or down to 543 K for the Pt-based glass-former, that is, slightly above their T_g to avoid sample crystallization. Below these temperatures, each sample were further cooled at variable cooling rates between q_c = 5000 and 0.2 K s^{-1} down to 183 K. After a 0.1 s isotherm at 183 K, an heat flow rate scan was recorded using a constant heating rate, up to above the melting point. Depending on the sample mass, a particular heating rate was chosen to maximize the signal to noise ratio and ranged from 100 K s^{-1} for the largest samples to 5000 K s^{-1} for the smallest (see Supplementary table 1). The program was looped each time utilizing a different cooling rate. Each heating scan was characterized by a recovery endotherm, with magnitude that increases with decreasing previously applied cooling rate, as a result of the glass relaxation on cooling. The Moynihan area matching method to calculate T_f (Ref. (86) of the main manuscript) results in the following equation:

$$\int_{T_f}^{T_1} (c_p^l - c_p^g) dT = \int_{T_2}^{T_1} (c_p - c_p^g) dT, \quad (1)$$

where c_p is the specific heat of the sample, c_p^l, c_p^g are the liquid and the glassy lines, T_1 and T_2 are

temperatures well above and below the T_g, respectively. For a reliable determination of c_p^l and c_p^g, we used data from step response analysis, delivering the reversing specific heat. As shown in panel b of Supplementary Figure 1, above all for c_p^l, in doing so a wide temperature range, about 60 K, is considered for the linear fitting.

The Moynihan method relies on the evaluation of the fictive temperature in heating scans, disregarding the cooling curves, that suffer from low sensitivity especially at low rates. However, the T_f determined on heating can be shifted upward due to overheating. This phenomenon is more pronounced in case of cooling rates exceeding the heating, and is caused by the glassy non-isothermal aging in the time spent during the heating ramp before devitrification takes place. To evaluate the magnitude of the overheating, as a showcase we performed a study obtained using a Au-based sample of the glass transition temperature signal on cooling and the fictive temperature in the consecutive heating both obtained with a scanning rate of 5000 K s^{-1}. This is reported in Supplementary Figure 1, where a perfect coincidence is found between T_g^{cool} (glass transition temperature during cooling), calculated at the half step of c_p, and the fictive temperature value T_f, obtained from the re-heating scan. The absence of superheating effects even in bulky samples is reported in Ref. (10) of the main manuscript.

Supplementary Note 2. Independence of vitrification kinetics in samples with identical l_{eq}. the $Au_{49}Cu_{26.9}Si_{16.3}Ag_{5.5}Pd_{2.3}$ at. % glass-former was mainly characterized by means of the Flash DSC 1 using UFS sensors. As a consequence of the wetting interaction of the glass with the aluminum membrane of the UFS sensor its resulting geometry was in the form of an irregular film,

as shown in the SEM micrographs reported in Supplementary Figure 4a. We complemented these measurements with the characterization of vitrification kinetics of a specimen of this composition having l_{eq} = 3.6 μm using the Flash DSC 2+ and a HTS sensor. Repeating melting of this specimen on the silicon nitride membrane of the HTS sensor did not result in the same wetting behavior that was observed with the UFS sensors, rather the specimen transformed its shape from a flake into an irregularly shaped sphere (see Supplementary Figure 4a). Supplementary Figure 4 shows in panel (b) the Flash DSC 2+ scans of this Au-specimen using the HTS sensor and in panel (d) the corresponding cooling rate dependent T_f values as a function of inverse temperature as yellow stars superimposed to the values obtained for the same composition with the UFS sensors and a Flash DSC 1. As can be observed in the latter panel, vitrification kinetics in the sample with l_{eq} = 3.6 μm measured by the Flash DSC2+ (yellow stars) perfectly matches with that of the sample with equal l_{eq} but measured in the Flash DSC 1 (green circles) even if the specimens exhibit different wetting behaviour thus different sample geometry, resulting in different heat flow rate scans (see panel (c) of Supplementary Figure 4). These results highlight the relevance of l_{eq} in determining vitrification kinetics, in line with the predictions of the FVHD model.
